# Utilization of Traditional Chinese Medicine Practitioners in Later Life in Mainland China

**DOI:** 10.3390/geriatrics4030049

**Published:** 2019-08-25

**Authors:** Jessica Yi Han Aw, Vasoontara Sbirakos Yiengprugsawan, Cathy Honge Gong

**Affiliations:** 1 Centre for Research on Ageing Health and Wellbeing (CRAHW), Research School of Population Health, The Australian National University, Canberra ACT 2601, Australia; 2 Australian Research Council Centre of Excellence in Population Ageing Research (CEPAR), University of New South Wales Business School, Kensington NSW2033, Australia

**Keywords:** traditional Chinese medicine (TCM), TCM practitioner use, older Chinese

## Abstract

Mainland China is one of the world’s most rapidly aging countries, and yet there is very limited literature on traditional Chinese medicine (TCM) use in older individuals. This study aimed to determine the national and provincial prevalence of TCM practitioner utilization in later life and associated factors. We used World Health Organization China Study on Global Aging and Adult Health Wave 1 data to determine descriptive statistics of the study population of participants aged 50 years and over. Multivariate logistic regression was conducted controlling for sociodemographic and health factors. A total of 14% of participants utilized a TCM practitioner, and the prevalence of utilization varied significantly by locality. Utilization was more likely in participants living in rural areas [adjusted odds ratio (OR) = 12.96; *p* < 0.001], Hubei (OR = 7.17; *p* < 0.001), or Shandong provinces (OR = 4.21; *p* < 0.001) and being diagnosed with chronic lung disease (OR = 1.97; *p* = 0.005). Hence, rurality, provincial influence, and chronic lung diseases are significant factors associated with TCM practitioner utilization among older individuals in China. These findings may inform policy for preservation and development of TCM nationally as well as its sustainability in an increasingly aging society.

## 1. Introduction

Healthy aging is an emerging agenda in many low and middle-income countries (LMICs) as the World Health Organization (WHO) estimates 80% of older individuals will be living in these countries by 2050 [1]. The aging population has placed greater strain on health services, especially for chronic diseases, necessitating the need for later-life health and medicine research [2]. In LMICs, traditional medicines and its practitioners are considered an important resource for population health by being perceived to be more affordable, accessible, and culturally acceptable to the communities when compared to modern medicine [3,4]. Several studies have demonstrated a high prevalence of traditional medicine utilization in older individuals of diverse ethnic backgrounds [3,5,6].

Mainland China is one of the world’s most rapidly aging countries as a result of both the one-child policy introduced in 1979 and increasing life expectancy [7]. The health care system of mainland China is unique in that western medicine (WM) and traditional medicine are practiced concurrently at all levels [8]. Traditional Chinese medicine (TCM) approaches are typically used for improving the immune system and prevention of chronic diseases, while WM is used for acute diseases. 

While WM and TCM are practiced concurrently, TCM and TCM practitioners tend to be more widely utilized in rural areas [9]. In Mao’s era of post-liberation, around 80% of the elderly were living rurally, predominantly agricultural farmers dependent on their children for financial support and care [10]. In the 1960s and 1970s, a free and basic public health system was introduced and built up in all rural areas where immunization services were free. The barefoot doctor program set up during this era was regarded, both inside and outside of China, as a low-cost solution built around easily available indigenous (Chinese herbal) medicines that were more affordable compared to WM. As there was a gradual increase in WM knowledge, WM was integrated into medical practice with TCM [11]. 

Throughout the 1970s, the decreasing prices of western pharmaceuticals resulted in a significant increase in pharmaceutical use in villages. When rural reform was initiated in the early 1980s, previously free basic public health care services became paid services, medical care in rural areas became under-resourced [11], and barefoot doctors became private medical practitioners. Because no fees were usually charged for consultations and examinations, village clinics became dependent on profits from pharmaceutical sales to maintain daily operations. Prescribing western pharmaceuticals became even more popular [11].

In 1999, the Chinese government introduced a licensing system for WM practitioners requiring up to 3 years of post-education clinical experience and a qualifying examination [12]. The same qualifying examination is required to be a licensed TCM practitioner [12]. In 2006, there were 166,614 TCM practitioners in hospitals and 7843 TCM practitioners in community health centers [12]. Today, TCM practitioners make up around 12% of all licensed doctors [12]. According to the national health statistics in China, the knowledge structure of candidates for the Excellent Village Doctor Award in China were 69% (Western) and 27.2% (Chinese) in 2005, which has changed to 73.5% (Western) and 25.5% (Chinese) in 2010, showing an increasing trend of village doctors with WM knowledge in rural China [13].

The 1980s saw major socioeconomic reforms where family size decreased, while medical costs and inequalities increased dramatically. Although the new rural cooperative medical insurance initiated in 2009 has covered most rural residents, reimbursement rates are still much lower than other medical insurances for employees [7]. Despite what has been argued as a cultural shift towards WM in the general population as a result of westernization and urbanization, the more affordable TCM and TCM practitioners remain widely utilized in rural areas, particularly for chronic diseases [14]. According to the China Health Statistics Yearbook 2018, there are 2641 million visits to Chinese traditional medicines (TCM) practitioners in the year 2017, which is about 32.3% of total visits to medical practitioners [15]. The number and proportion of visits to TCM practitioners in China had increased from 1945 million and 28.2% in the year 2012 to 2642 million and 32.3% in the year 2017, respectively [15] (for more information, see Appendix A Table A1).

Despite a 2500-year history of TCM use, studies assessing national TCM utilization in mainland China are very limited [9,16]. Furthermore, only one national study has focused on older individuals, using data from China Health and Retirement Longitudinal Study (CHARLS) to estimate 19.3% of the population utilizes TCM [16]. In regional areas around mainland China, varying prevalence have also been recorded [17,18]. A systematic review also found that perception of TCM varies between regions, with some viewing TCM as a basis for self-care and others as a method of symptom control and recuperation or synergistic use with WM [4]. While these studies may suggest the significance of regional differences in TCM utilization, as of yet, no national study, including different regions, has assessed this.

The World Health Organization China Study on global Aging (WHO SAGE China) Wave 1 provides a nationally representative dataset focusing on individuals 50 years and older. One study has previously utilized SAGE China 2007 survey to estimate the national prevalence of traditional medicine practitioner utilization and associated factors for individuals 18 years and older [9]. 

Utilizing the same data from WHO SAGE China 2007 survey, this study focused on later life and aimed to assess both the national and provincial specific prevalence of TCM practitioner utilization as well as its associated chronic diseases, sociodemographic and health factors in mainland China. These findings may help to inform the local implementation of the WHO Traditional Medicine Strategy 2014–2023, which aims to develop a policy to sustain the role of traditional medicine [19,20]. These findings may also more broadly inform the emerging agenda of healthy aging in other LMICs.

## 2. Materials and Methods 

### 2.1. Data

The data was obtained from the World Health Organization China Study on Global Aging and Adult Health Wave 1 (SAGE China). A clustered household sampling strategy was used for the national representation of older participants aged 50 years and over where 10,278 households across 8 main provinces in China were selected for the survey. The selection of the 8 provinces: Guangdong, Hubei, Jilin, Shaanxi, Shandong, Shanghai, Yunnan, and Zhejiang was determined through three steps. Firstly, 31 provinces were divided into eastern middle and western areas according to geography and socioeconomic level. Next, four provinces were randomly selected from the eastern, two from the central, and two from western areas. Finally, one country from rural national Death Surveillance Points (DSPs) and one district from urban DSPs in each province were selected.

All 50+ participants from each household were surveyed, while only one 18–49-year-old participant was surveyed for comparison purposes. The SAGE China survey consisted of a total of 14,482 consenting participants from 8 main provinces in China, surveyed from 2007 to 2010 (1676 reported aged under 50 years, and 12,806 reported aged 50 and over). 

Our analysis focused on respondents aged 50 and over with reported information on using a TCM practitioner in last 12 months (n = 5981) as they are more likely to be nationally representative from which the study populations were drawn. As a result, 6798 participants aged 50 and over were omitted due to not responding to questions about health care providers. This selection also avoided any bias in our results from the very small sample size and biased weighting up for the group aged 18–49 years in SAGE data (only 688 individuals aged 18–49 completed information on utilization of a TCM practitioner). The SAGE China study received human subjects testing and ethics council approval from the research review boards local to each participating site and the WHO Ethical Review Committee. Written informed consent was obtained from each respondent before the interview and examination. A standard consent form, approved by the WHO ethics review committee, was read to the respondent in the respondent’s language [9].

### 2.2. Covariates

We followed the Andersen Healthcare Utilization Model [21] and literature [9] to control for predisposing factors (age, gender, marital status), enabling factors (education, income quintile, rurality, provinces), and need (self-reported health, chronic diseases, smoking, and drinking behaviors). However, due to a high correlation between self-reported health and the prevalence of chronic diseases, we omitted self-reported health from our final modeling analysis. Health covariates were assessed in the variables of alcohol use, tobacco use at time of the interview, and diagnosis of several major chronic diseases [9,16,17]. Every covariate had a ‘don’t know’ or ‘not applicable’ response options, which were omitted from the analysis.

#### 2.2.1. Demographics 

Locality was classified as either living in an urban or rural region. Provinces were classified into Guangdong, Hubei, Jilin, Shaanxi, Shandong, Shanghai, Yunnan, and Zhejiang. Age was grouped into 50–64 and 65 years and over, where 65 and over was used to further classify older individuals who may present with more health complications. Marital status was classified as, ‘never married’, ‘currently married/cohabiting’, ‘separated/divorced’, and ‘widowed’. Income quintiles were assigned to participants as 1 (poorest) to 5 (wealthiest) based on the permanent income of all participants. Education was classified into the participant’s highest level of education being ‘primary/less’, ‘secondary’, or ‘tertiary/more’. 

#### 2.2.2. Health and Health Behaviors 

Diagnosed chronic diseases in this study include angina, asthma, arthritis, cataracts, chronic lung disease [emphysema, bronchitis, chronic obstructive pulmonary disease (COPD)], depression, diabetes, hypertension, or stroke. Each of the diseases was classified as ‘yes’ the participant had been diagnosed for that disease, or ‘no’ they had not been diagnosed. Alcohol use and tobacco use were both classified as the participant having used or not having used alcohol/tobacco in their life. 

#### 2.2.3. Definition of TCM Use

TCM includes several aspects from consulting a TCM practitioner to taking traditional Chinese herbs, herbal products, and traditional therapies [16,22]. Previous studies have defined TCM use in the context of the data collected. Some studies have defined TCM use broadly including the consumption of traditional Chinese herbs, herbal products, and utilization of therapies, such as acupuncture, massage, and cupping [16,17,22,23,24]. Other studies have defined TCM use with regards to visiting a TCM health care provider, such as a TCM practitioner [9,18,25,26]. Due to the available data in SAGE collected in this study, the use of TCM in this study was defined concerning a visit to a TCM practitioner (中医医生) in the most recent three visits of health care providers in the past 12 months. 

In SAGE China survey, participants were asked 3 questions regarding the type of health care providers they had visited in the past 12 months. In the first question, participants were asked: “Which was the last (most recent) health care provider you visited?”. The following two questions asked which health care provider was visited in the second and third most recent visits. The response options for these three questions were “medical doctor”, “nurse/midwife”, “dentist”, “physiotherapist or chiropractor”, “Traditional Medicine practitioner”, “pharmacist, druggist”, “home health care worker”, or “don’t know”.

Utilization of a TCM practitioner was classified as any participant that provided the ‘TM practitioner’ response for any of these 3 questions. Non-utilization of a TCM practitioner was classified as any participant that did not provide the ‘TM practitioner’ response to all of the 3 questions. Participants who did not respond to all three questions were excluded from analysis as we were unable to determine whether or not they utilized a TCM practitioner.

### 2.3. Statistical Analysis

Statistical analysis was conducted in Stata software version 14 (StataCorp LP) and R studio version 1.1.423. The "post stratified household weight" (pweight) from the survey data was used for our analysis as all members aged 50 and over in the selected households were surveyed so that the household weight is equal to the individual weight for respondents aged 50. 

To characterize TCM practitioner utilization within the study population, descriptive statistics were produced for utilization of and non-utilization of a TCM practitioner. To assess the association between covariates and utilization of a TCM practitioner, the univariate and multivariate analyses were conducted. Logistic regression was used for a binary outcome: utilization or non-utilization of a TCM practitioner. In univariate analysis, each covariate was assessed individually where an odds ratio (unadjusted OR) was produced for each non-reference level, and their significance levels are reported in Table 1. In multivariate analysis, Model 1 included sociodemographic covariates and locality (both urban/rural residence and provinces) but excluded health covariates (health behaviors and chronic diseases); Model 2A included all covariates except for province, and Model 2B included all covariates (as our final model). The inclusion and exclusion of different variables in different models enabled us to check the robustness of our results.

## 3. Results

### 3.1. Descriptive Statistics

The characteristics of 5981 participants aged 50 and over are summarized in Table 1. A total of 14% of the participants utilized a TCM practitioner. No significant differences in the prevalence of TCM practitioner utilization between sex or age groups were observed. A significantly higher prevalence was observed in participants that never married (31.3%) compared to other marital statuses. Prevalence of TCM practitioner utilization was similar between income quintiles one to four, which were all significantly higher than in the wealthiest quintile five (4%). Significantly higher prevalences were seen in primary (17%) and secondary educated (9.3%) participants compared to those tertiary educated (2.4%). 

Rural regions also had a significantly higher prevalence of TCM practitioner utilization (22.9%) compared to urban regions (2.6%). Large variations were observed between provinces. Significantly higher prevalences were found in Hubei (33.6%) and Shandong (25.4%) when compared to participants for Guangdong (3.3%). Significant differences in the prevalence of TCM practitioner utilization were seen in participants that used tobacco (15.7%), and no significant differences were seen in those that used alcohol. 

Amongst diagnosed chronic diseases, there was a significantly lower prevalence of TCM practitioner utilization in participants diagnosed with angina (9.8%), arthritis (12%), cataracts (6.2%), diabetes (7.6%), and hypertension (11.7%). Prevalence of TCM practitioner utilization was slightly higher for participants with chronic lung disease diagnosis, but this did not reach significance at the univariate level.

### 3.2. Multivariate Analysis

When controlling for all sociodemographic covariates in Model 1 of the multivariate analysis (Table 2), only income quintile, locality, and province covariates were significant. Living in a rural region [odds ratio (OR) = 13.54; *p* < 0.001], or in Shandong or Hubei provinces, was highly significantly associated with TCM practitioner utilization (ORs ranging from 3.65 to 6.67; *p* < 0.001), while TCM practitioner utilization was significantly less associated with living in Shaanxi, Yunnan, or Zhejiang provinces (ORs ranging from 0.16 to 0.26; *p* ≤ 0.002). 

Given the significance of the provincial covariate, Model 2A further controlled for health covariates but excluded the provincial covariate. Locality and income quintile remained significant. Additionally, being female (OR = 1.49; *p* = 0.03) and in the relatively older age group of 65 years and over (OR = 1.49; *p* = 0.006) gained significance, and being diagnosed with chronic lung disease (OR = 1.76; *p* = 0.007) was significant in TCM practitioner utilization.

In Model 2B of Table 2, controlling for all covariates, age group, sex, and income quintiles lost significance, while rurality remained highly significant (OR = 12.96; *p* < 0.001). Living in Hubei (OR = 7.17; *p* < 0.001) or Shandong (OR = 4.21; *p* < 0.001) was significantly associated with higher TCM practitioner utilization, while living in Shaanxi (OR = 0.27; *p* < 0.001), Yunnan (OR = 0.24; *p* = 0.004), or Zhejiang (OR = 0.16; *p* = 0.001) was significantly associated with lower TCM practitioner utilization, and this was consistent to the estimates in Model 1. Among the disease diagnoses, having chronic lung disease was the only diagnosis that remained significantly associated (OR = 1.97; *p* = 0.005) with higher TCM practitioner utilization. No significant association was found between TCM practitioner utilization and smoking or drinking behaviors.

### 3.3. Robustness Check

A robustness check was conducted to examine whether the statistical significance of provincial differences in TCM practitioners use was influenced by smaller sample sizes in Yunnan (n = 14), Zhejiang (n = 14), and Jilin (n = 5). To overcome the issue of smaller sample size, higher-income provinces Guangdong, Shanghai, and Zhejiang and lower-income provinces Jilin, Shaanxi, and Yunnan were grouped. Model 2B of Table 2 was run again but replacing the original province covariate with Hubei, Shandong, and the two grouped provinces. The coefficient estimates for Hubei and Shandong remained robust (OR = 13.34 and 7.73, respectively) when compared to the estimated coefficients of model 2B in Table 2 (OR = 7.17 and 4.21, respectively).

To understand which underlying aspects may contribute to the significance of these provincial differences, the descriptive analysis of the urban and rural regions of Hubei, Shandong, and the two grouped provinces was conducted (Table 3). The highest proportions of TCM practitioner utilization were observed in rural Hubei and rural Shandong (57% and 39.5%, respectively) when compared to other areas (less than 6%). These regions also had higher proportions of tobacco use (46.4% and 41.2%, respectively) compared to the rural areas of the grouped provinces (less than 40%). Rural Hubei had a higher proportion of older individuals in the lowest two income quintiles, while urban Hubei and rural Shandong had a higher proportion of older people in quintiles three and four. Additionally, Hubei and the grouped low income provinces (Jilin, Shaanxi, and Yunnan) had a higher proportion of chronic lung diseases in both urban and rural areas (8.8% in rural Hubei, and 11% and 8.2% in urban and rural areas of the grouped low income provinces when compared to 8.5% and 6.9% in the urban and rural areas of the grouped high income provinces). The higher proportion of using tobacco is likely to lead more lung diseases (such as chronic obstructive pulmonary disease (COPD)), for which the traditional Chinese medicines were perceived to be more effective than the western pharmaceuticals (such as for coughing, having the flu or fever, etc.). 

## 4. Discussion

This study estimated that nationally, 14% of individuals aged 50 years older utilized a TCM practitioner. This prevalence varied between urban and rural residence, across provinces, as well as by age, income quintile, and the type of chronic diseases. Individuals that utilize TCM practitioners were more likely to live in rural areas and be diagnosed with certain chronic diseases, and these were consistent with the findings of a previous study [9]. However, the significance at the provincial level, including Shandong or Hubei provinces, has not been previously assessed.

Only two studies have previously reported national statistics of TCM utilization in mainland China [9,16]. Oyebode et al. (2016), also using the SAGE China dataset, estimated a lower 9.4% national prevalence, and this difference is likely attributable to our analysis focusing on older populations aged 50 and over [9]. Liu et al. (2015), using CHARLS data, had a higher estimate of 19.3% for individuals 45 and older. However, TCM utilization was defined more broadly including personal use of TCM herbs, herbal products, and practices additional to practitioner utilization, such as moxibustion, and Chinese massage [16], while such data was not collected in the SAGE China dataset. 

The prevalence of TCM practitioner utilization varied significantly between provinces, and this was further supported by participants from some provinces being more likely to utilize a TCM practitioner compared to others. Rurality also remained significant across all analyses consistent with the findings of Oyebode et al. (2016) [9]. There are known disparities in the self-reported health and health outcomes between urban and rural provinces in mainland China [27]. These disparities may be reflective of fewer specialized WM healthcare options in rural areas, resulting in most health treatment administered by TCM practitioners, stemming from the historical use of TCM as a low-cost alternative in mending a broken healthcare system in rural areas [10,28]. These disparities could also be, in part, explained by poorer health behaviors in terms of tobacco use, alcohol consumption, diet, and physical inactivity observed in rural residents compared to urban residents [29]. Our robustness tests confirmed that there were higher proportions of tobacco use in Hubei and Shandong areas, which are also regions with major tobacco planting areas. These local contexts may be important in explaining our finding of the significance of chronic lung diseases in TCM practitioner utilization, and this was consistent with Liu et al.’s (2015) findings as TCM is preferred over WM in treating chronic conditions due to fewer side effects [4,16]. Chronic lung disease has also been well defined in TCM practice [30].

Before controlling for province, the relatively older group aged 65 and over were more likely to utilize a TCM practitioner when compared to those aged 50 to 64. This is consistent with the current literature [9,16]. Income quintile three and four were also significant before controlling for province, which is consistent with a study that suggested relatively higher socioeconomic districts within Hubei province were associated with higher TCM use [25]. Although education and sex were found to be significantly associated with TM use for Beijing, Taiwan, and Hong Kong in literature [13,14,19,21], we did not find significant associations for these covariates after controlling for other socioeconomic, health, and locality variables at later life in China. 

To the best of our knowledge, this is the first study to assess TCM practitioner utilization at the provincial level in mainland China. This enabled identification of varied usage patterns of TCM practitioner utilization and suggested the need for more representative samples at provincial levels. This study also more broadly addressed an important gap in the literature regarding traditional medicine utilization in older individuals given previous studies demonstrate a high prevalence of utilization [3,4,5]. While Oyebode et al. (2016) also used SAGE China survey data, they focused on all age groups and controlled for both self-reported health and chronic diseases in their logistic regression model, and sex and education were not included in their models [9]. This study improved estimations by including sex and education while excluding self-reported health from the multivariate regression as older individuals with chronic diseases are generally more likely to report bad health [7]. 

Despite these improvements, there are several limitations to this study. Firstly, the sample size for TCM practitioner utilization in more than half of the provinces were too small to run multivariate analyses to find meaningful factors associated with utilization at the provincial level. While some provinces had a smaller sample size that utilized TCM practitioner, our robustness check enabled confidence in the significance of higher utilization of TCM practitioners in both Hubei and Shandong provinces, while our estimations for other provinces needed to be interpreted with caution due to small sample sizes. Since data was collected via interviews, respondent bias might be present in aspects, such as depression diagnosis. Given mental health in China is highly stigmatized [31], there may likely have been underreporting or undiagnosed participants in our dataset on the prevalence of depression. A large-scale national study focused on mental health found that a higher statistic of 2.4% of participants had depressive symptoms [32], while in our study, less than 1% of all participants were diagnosed. 

Furthermore, the data collected limited our definition of TCM use to visiting or not visiting TCM practitioners in the participants’ three most recent health care provider visits in the past 12 months. In mainland China, often a mix of TCM as well as WM is utilized [12,33]. Additionally, in a study of outpatients at Fudan University’s Shanghai Cancer Center, the most common form of TCM use was the use of tonics and Chinese herbal products, which can be sourced independently of visiting a TCM practitioner [4,23,34]. TCM practitioner utilization may also be inadequately captured in the three most recent health care provider visits defining our study population. Consequently, the national statistic this study estimates may underestimate both the true prevalence of TCM practitioner utilization and TCM use. Another limitation was that the data did not include reasons for TCM practitioner utilization that may have provided further insight into covariate associations. 

To preserve and promote TCM, the Chinese government has set a trajectory that by 2020 TCM health services will be accessible to every Chinese citizen and by 2030, TCM health services will provide for all medical needs [35]. The integrated licensing system and utilization of TCM and WM in China could inform the integration of traditional medicine and WM in other LMICs [10,12,14]. However, the clinical efficacy of the integrated approach using WM and traditional approaches to prevent and treat diseases need to be further investigated and evaluated carefully.

Our data have demonstrated the significance of locality and population sub-groups in TCM practitioner utilization, suggesting a need for the broader data collection on individual utilization of TCM (the utilization of TCM practitioner, herbs, herbal products, and traditional therapies) by both residence and individual characteristics. These local contexts may also reflect varied access to different health care options as well as local cultural contexts in terms of health behaviors like smoking, which may be important areas for primary prevention of chronic disease in elderly and warrants further research. Collection and assessment of such data can thereby target health policy to achieve these trajectories. In doing so, this may further enhance the ability of other LMICs to better integrate local traditional medicine into their health care systems in accordance with the WHO’s Traditional Medicine Strategy [19].

## 5. Conclusions

This study found rurality, province, and chronic lung disease diagnosis as significant factors associated with TCM practitioner utilization among older individuals in China. These factors may reflect structural differences in access to health care between urban and rural regions of different provinces and also suggest the importance of local cultural context in the utilization of a TCM practitioner. These findings may inform TCM preservation and development, health interventions, and integration with WM to improve the health and health service use at later life in an aging society. 

## Figures and Tables

**Table 1:** Characteristics of participants that utilized and did not utilize TCM (traditional Chinese medicine) practitioners, World Health Organization (WHO) China Study on Global AGEng and Adult Health (SAGE) (Wave 1), surveyed from 2007 to 2010.

Characteristics	Proportion by Characteristics % (N)		Prevalence of TCM Users %	
	TCM Practitioner Utilization (n = 559)	non-TCM Practitioner Utilization (n = 5422)		
**Overall**			14	
Sex				
Male (Ref)	258	2393	13.9	
Female	301	3029	14	
Age group				
50–64 (Ref)	321	3136	14.4	
65+	238	2286	13.2	
Marital Status				
Never married	13	42	31.3	**
Currently married/cohabiting (Ref)	465	4454	14	
Separated/divorced	4	103	8	
Widowed	77	822	12.9	
Income quintile				
1 (poorest)	112	919	15.3	***
2	120	966	16.3	***
3	129	1041	17.6	***
4	142	1204	16.9	***
5 (wealthiest) (Ref)	48	1271	4	
Education				
Primary or less	258	1953	17	***
Secondary	112	1842	9.3	*
Tertiary or more (Ref)	7	298	2.4	
Locality				
Urban (Ref)	83	2769	2.6	
Rural	476	2653	22.9	***
Province				
Guangdong (Ref)	28	775	3.3	
Hubei	170	547	33.6	***
Jilin	5	318	1.6	
Shaanxi	45	872	3.8	
Shandong	242	876	25.4	***
Shanghai	41	931	5	
Yunnan	14	497	2.7	
Zhejiang	14	606	2.1	
Health Risk Behaviors				`
Tobacco				
Used tobacco	218	1754	15.7	*
Never used tobacco (Ref)	340	3668	12.9	
Alcohol				
Used alcohol	197	1709	14.8	
Never used alcohol (Ref)	361	3706	13.5	
Disease Diagnosis				
Angina				
Yes	37	558	9.8	*
No (Ref)	521	4851	14.4	
Asthma				
Yes	10	164	8.2	
No (Ref)	549	5232	14.2	
Arthritis				
Yes	140	1491	12	*
No (Ref)	419	3930	14.7	
Cataracts				
Yes	31	619	6.2	***
No (Ref)	523	4721	14.9	
Chronic lung disease				
Yes	68	588	14.6	
No (Ref)	490	4830	13.9	
Depression				
Yes	2	556	16.3	
No (Ref)	21	5394	13.9	
Diabetes				
Yes	23	495	7.6	**
No (Ref)	535	4918	14.6	
Hypertension				
Yes	193	1793	11.7	*
No (Ref)	383	3588	14.9	
Stroke				
Yes	21	254	11.6	
No (Ref)	538	5166	14.1	

Note: Prevalence of TCM practitioner utilization; * indicates *p*-value ≤ 0.05, ** *p*-value ≤ 0.01, *** *p*-value ≤ 0.001, Ref denotes reference level.

**Table 2:** Multivariate regression results on the utilization of TCM practitioners, WHO SAGE China (Wave 1).

	Multivariate Logistic Regression: Adjusted Odds Ratios (OR)								
	Model 1		Model 2A		Model 2B				
	OR	*p*-value	OR	*p*-value	OR	*p*-value			CI
Sex									
Male (Ref)									
Female	0.98	0.883	1.49	0.030	1.32		0.194		0.87–2.01
Age group									
50–64 (Ref)									
65+	1.20	0.243	1.49	0.006	1.33		0.079		1.33–1.84
Marital Status									
Never married	0.87	0.857	2.32	0.173	1.09		0.908		0.26–4.51
Currently married/cohabiting (Ref)									
Separated/divorced	0.69	0.571	0.63	0.455	0.61		0.435		0.18–2.10
Widowed	0.95	0.858	0.84	0.498	0.84		0.514		0.49–1.43
Income quintile									
1 (poorest)	1.08	0.817	0.94	0.833	1.07		0.836		0.57–2.02
2	0.99	0.983	1.17	0.524	0.95		0.871		0.54–1.69
3	1.38	0.222	2.05	0.002	1.34		0.263		0.80–2.24
4	1.61	0.043	2.31	<0.001	1.58		0.053		0.99–2.50
5 (wealthiest) (Ref)									
Education									
Primary or less	1.24	0.688	0.93	0.884	1.20		0.743		0.40–3.47
Secondary	0.92	0.874	0.81	0.688	0.86		0.771		0.31–2.40
Tertiary or more (Ref)									
Locality									
Urban (Ref)									
Rural	13.54	<0.001	13.30	<0.001	12.96		<0.001		8.24–20.4
Province									
Guangdong (Ref)									
Hubei	6.67	<0.001			7.17		<0.001		3.82–13.4
Jilin	1.15	0.814			1.21		0.762		0.35–4.26
Shaanxi	0.26	<0.001			0.27		<0.001		0.13–0.56
Shandong	3.65	<0.001			4.21		<0.001		2.24–7.89
Shanghai	0.80	0.547			0.92		0.819		0.43–1.93
Yunnan	0.23	0.002			0.24		0.004		0.09–0.64
Zhejiang	0.16	0.001			0.16		0.001		0.05–0.49
Health Risk Behaviors									
Tobacco									
Used tobacco			0.82	0.275	0.80		0.276		0.53–1.20
Never used tobacco (Ref)									
Alcohol									
Used alcohol			1.01	0.935	0.93		0.678		0.65–1.32
Never used alcohol (Ref)									
Disease Diagnosis									
Angina									
Yes			0.85	0.494	0.65		0.112		0.38–1.11
No (Ref)									
Asthma									
Yes			0.50	0.192	0.48		0.194		0.16–1.45
No (Ref)									
Arthritis									
Yes			0.91	0.516	0.88		0.429		0.64–1.21
No (Ref)									
Cataracts									
Yes			0.73	0.331	0.98		0.952		0.51–1.89
No (Ref)									
Chronic lung disease									
Yes			1.76	0.007	1.97		0.005		1.23–3.15
No (Ref)									
Depression									
Yes			2.38	0.423	3.38		0.133		0.69–16.5
No (Ref)									
Diabetes									
Yes			0.76	0.372	0.62		0.150		0.32–1.19
No (Ref)									
Hypertension									
Yes			0.97	0.856	0.86		0.366		0.62–1.19
No (Ref)									
Stroke									
Yes			1.01	0.972	0.95		0.892		0.47–1.94
No (Ref)									

Notes: Ref denotes reference level, OR = adjusted odds ratio, CI = 95% odds ratio confidence interval. Model 1 included sociodemographic covariates and locality (urban/rural residence and province) but excluded health covariates (health behaviors and chronic diseases). Model 2A included all covariates except province dummies. Model 2B included all covariates as the final model.

**Table 3:** Proportions of respondents by provinces and major characteristics.

	(1) Guangdong, Shanghai, and Zhejiang		(2) Hubei		(3) Shandong		(4) Jilin, Shaanxi, and Yunnan	
	Urban	Rural	Urban	Rural	Urban	Rural	Urban	Rural
% Overall	49.8	50.2	47.5	52.5	49.3	50.7	49.2	50.8
% of TCM Practitioner utilization	1.5	5.7	5	57	1.3	39.5	5.6	2.6
Income quintile								
% of quintile 1	4.5	31.6	15.6	34.8	0.3	5.1	25	28.6
% of quintile 2	8.7	20.3	19.2	30.7	0.6	19	21.9	32.3
% of quintile 3	18.4	14.4	23.2	15.6	9.1	34.2	27.1	19.8
% of quintile 4	25	15.6	26.5	14	29.6	36.7	18.7	13.4
% of quintile 5	43.4	18.1	15.4	4.9	60.3	5	7.3	5.9
Tobacco								
% using tobacco	26.1	38	37.6	46.4	17	41.2	31.1	36.3
Chronic lung disease								
% diagnosed	8.5	6.9	15.7	8.8	6.6	4.9	11	8.2

Note: Proportions of the study population of consenting individuals 50 years and over (n = 12,806) by urban and rural province groups of Guangdong, Shanghai, and Zhejiang; Hubei; Shandong and Jilin, Shaanxi, and Yunnan.

## Appendix A

**Table A1:** Number of visits to medical practitioners in China, 2012–2017.

	2012	2013	2014	2015	2016	2017
Visits to Chinese traditional medicines (TCM) practitioners (1,000,000)	1945	2110	2280	2367	2501	2642
Total visits to practitioners (1,000,000)	6888	7314	7602	7693	7932	8183
Proportion of TCM practitioners (%)	28.2%	28.9%	30%	30.8%	31.5%	32.3%

Data source: The China Health Statistics Yearbook 2018 [15].
